# Surgical outcomes of patients with unruptured anterior vs. inferior circulation aneurysms: A meta‑analysis

**DOI:** 10.3892/mi.2023.129

**Published:** 2023-12-28

**Authors:** George Fotakopoulos, Ioannis G. Lempesis, Vasiliki Epameinondas Georgakopoulou, Nikolaos Trakas, Pagona Sklapani, Konstantinos Faropoulos, Kostas N. Fountas

**Affiliations:** 1 Department of Neurosurgery, General University Hospital of Larissa, 41221 Larissa, Greece; 2 Department of Pathophysiology, National and Kapodistrian University of Athens, 11527 Athens, Greece; 3 Department of Biochemistry, Sismanogleio Hospital, 15126 Athens, Greece; 4 Department of Neurosurgery, Nicosia General Hospital, 2029 Nicosia, Cyprus

**Keywords:** unruptured aneurysm, anterior circulation aneurysm surgery, posterior circulation aneurysms surgery, unruptured anterior vs. posterior circulation aneurysms surgery

## Abstract

The treatment option for unruptured intracranial aneurysms (UIAs) depends on their natural history-related risk of rupture vs. the risk of surgical management. The present meta-analysis sought to assess the association between the surgical outcomes of anterior and posterior circulation UIAs. The present study investigated the comparative articles involving the surgical treatment of anterior vs. posterior circulation UIAs through electronic databases, including the Cochrane Library, PubMed (1980 to March, 2023), Medline (1980 to March, 2023) and EMBASE (1980 to March, 2023). Quoting all exclusion and inclusion criteria, nine articles finally remained for statistical analysis. The entire number of patients included in these nine articles was 3,253 (2,662 in the anterior and 591 in the posterior circulation UIAs group). The present meta-analysis proposes that the surgical treatment of anterior circulation UIAs is associated with better outcomes compared with the surgical management of posterior circulation UIAs.

## Introduction

Intracranial aneurysms are abnormal, balloon-shaped dilations of the walls of intracranial arteries. Depending on their size and other risk factors, such as cigarette smoking and an uncontrollably high blood pressure, they have a tendency to rupture.

Unruptured intracranial aneurysms (UIAs) are comparatively frequent lesions that account for 0.4-6% of the general population (1,2). During the previous decades, there was a huge debate on whether to treat UIAs or follow them up. On the one hand, the possible complications of the ‘wait and see’ approach, namely the rupture of the aneurysm (electrolyte disturbance, hydrocephalus, vasospasm, coma and mortality) had to be taken into account, while on the other hand, the possible complications of the elective surgical treatment of an intracranial aneurysm (post-operative pain, blood loss, epileptic seizures, cerebral laceration, neurological deficit and mortality) had to be considered. The ‘compass’ that was used to provide guidance of cases of UIAs was the annual rupture risk of a UIA vs. the risks associated with surgical management (3).

The annual risk of rupture during the lifetime of a patient with a UIA (also known as the natural risk) of UIAs is found to be 1-2%, and that risk is added to the risk of the following year for every year of life. Thus, for a 20-year-old patient with a UIA, there is a 40-80% chance of an aneurysm rupture by the age of 60 years, while for a 40-year-old patient with a UIA, there is a 20-40% chance of an aneurysm rupture by the age of 60 years. Additionally, the mortality of rate of patients with a ruptured aneurysm is 40%, while in other research series, that number increases to 50% (3).

By contrast, the morbidity associated with the microsurgical treatment of UIAs has been found to be lower than that for ruptured aneurysms (4,5). Under that scope, the suggested modality for UIAs was to treat them, as the treatment has superior results and fewer complications compared to the natural history of the disease and the possible complications following an aneurysm rupture, at least for the younger patients (4,5).

That dogma is used mostly for anterior circulation aneurysms, while the management method of a posterior circulation aneurysm is a debatable theme. The issue is that the majority of studies which mention outcomes from the surgical management of UIAs have excluded posterior circulation aneurysms, possibly since these aneurysms are considered surgically challenging and are associated with a higher morbidity risk compared with their anterior circulation counterparts (3-5). In detail, some reports mention a 4.2% unfavorable outcome rate associated with the surgical management of posterior circulation aneurysms. Notwithstanding, it should be noted that these reports included only giant aneurysms (3,4,6), which are the most demanding when they are treated surgically.

It is well known that there are some studies with notable findings; these studies evaluated the natural risk of bleeding in the UIAs and proposed various management options for posterior and anterior circulation aneurysms (7,8). However, there is limited information available regarding the specific influence of the location of the aneurysm (anterior vs. posterior circulation UIAs) on surgical outcomes.

The present meta-analysis aimed to assess the association between the surgical outcomes of patients with anterior vs. posterior circulation UIAs. Moreover, in order to define the procedural good neurological outcomes, morbidity and mortality, the modified Rankin scale (mRS) >2 was used for patients with a UIA that were treated surgically.

## Data and methods

### Literature search strategy

The present meta-analysis investigated the proportional articles on the surgical treatment of anterior vs. posterior circulation UIAs through electronic databases, including the Cochrane Library, PubMed (1980 to March, 2023), Medline (1980 to March, 2023) and EMBASE (1980 to March, 2023). Preferred Reporting Items for Systematic Reviews and Meta-Analyses (PRISMA) (9) served as the foundation for the protocol and manuscript design. In the Medical Subject Headings (MeSH) list, the following key words were used: ‘Anterior and posterior circulation aneurysms’, ‘unruptured aneurysms’, ‘anterior vs. posterior circulation aneurysm surgery’ and ‘unruptured aneurysm surgery’.

### Inclusion and exclusion criteria

All studies included in the present meta-analysis met the PICOS criteria as follows: i) Population: Limited to patients that underwent aneurysm clipping surgery for UIAs anterior and posterior circulation; ii) Intervention: Surgical treatment for UIAs; iii) Comparison: The outcomes were evaluated and compared; iv) Outcome measures: One of the primary outcomes, such as procedural morbidity (mRS >2), mortality and good neurological outcomes related to aneurysmal surgical treatment, were all evaluated. To shun publication bias, the concluding intent was to assemble a homogenous sum of studies involving only articles that evaluate only two modalities: A comparison between the surgical treatment of anterior and posterior UIAs. The present study excluded all articles that were reviews, editorials and case reports. Moreover, articles that investigated the pediatric population, unrelated outcomes, comorbidities, novel techniques in the experimental stage, or one of the two treatment options, and all those that revealed mixed or uncertain results, being divided between anterior and posterior circulation UIAs surgical treatment, were also excluded.

### Data extraction and outcome definition

Two authors (GF and KF) separately extracted data from the contained articles according to the epidemiology guidelines of the meta-analysis. The following critical information was retained: The main authors, publication year, entire number of cases in the anterior and posterior circulation UIA groups, outcome indicator, study type, etc. According to the Cochrane Handbook, the pulled-out data was entered into a planned, standardized table (https://training.cochrane.org/handbook).

In the case of a discrepancy, an additional author with authority made the concluding decision. Post-operative outcomes declared in the final pool articles were assessed at least 6 months following surgical treatment (UIAs, anterior or posterior circulation). Furthermore, to diminish the risk of bias in the articles, a quality assessment tool (the Newcastle-Ottawa Scale) was performed (Table I) (10). In addition, the patients were divided into two groups as follows: Those with anterior circulation UIAs and those with posterior circulation UIAs.

### Statistical analysis

All analyses were carried out using Review Manager Software (RevMan), version 5.4 (https://training.cochrane.org/online-learning/core-software/revman). Heterogeneity across trials was identified using I^2^ statistics; I^2^ >50% was considered high heterogeneity. A meta-analysis was conducted using a random-effect model according to the Cochrane Handbook for Systematic Reviews of Interventions (version 5.1.0; https://training.cochrane.org/online-learning/coresoftware/revman); or else, the fixed-effect model was carried out. The continuous outcomes (procedural morbidity (mRS >2), mortality and good neurological outcome related to aneurysmal surgical treatment) were stated as a weighted mean difference with 95% confidence intervals (CIs). In the case of discontinuous variables, odds ratios (ORs) with 95% CIs were obtained for the evaluation. A P-value <0.05 was considered to indicate a statistically significant difference.

## Results

### Studies in the final pool

**Figure 1:**
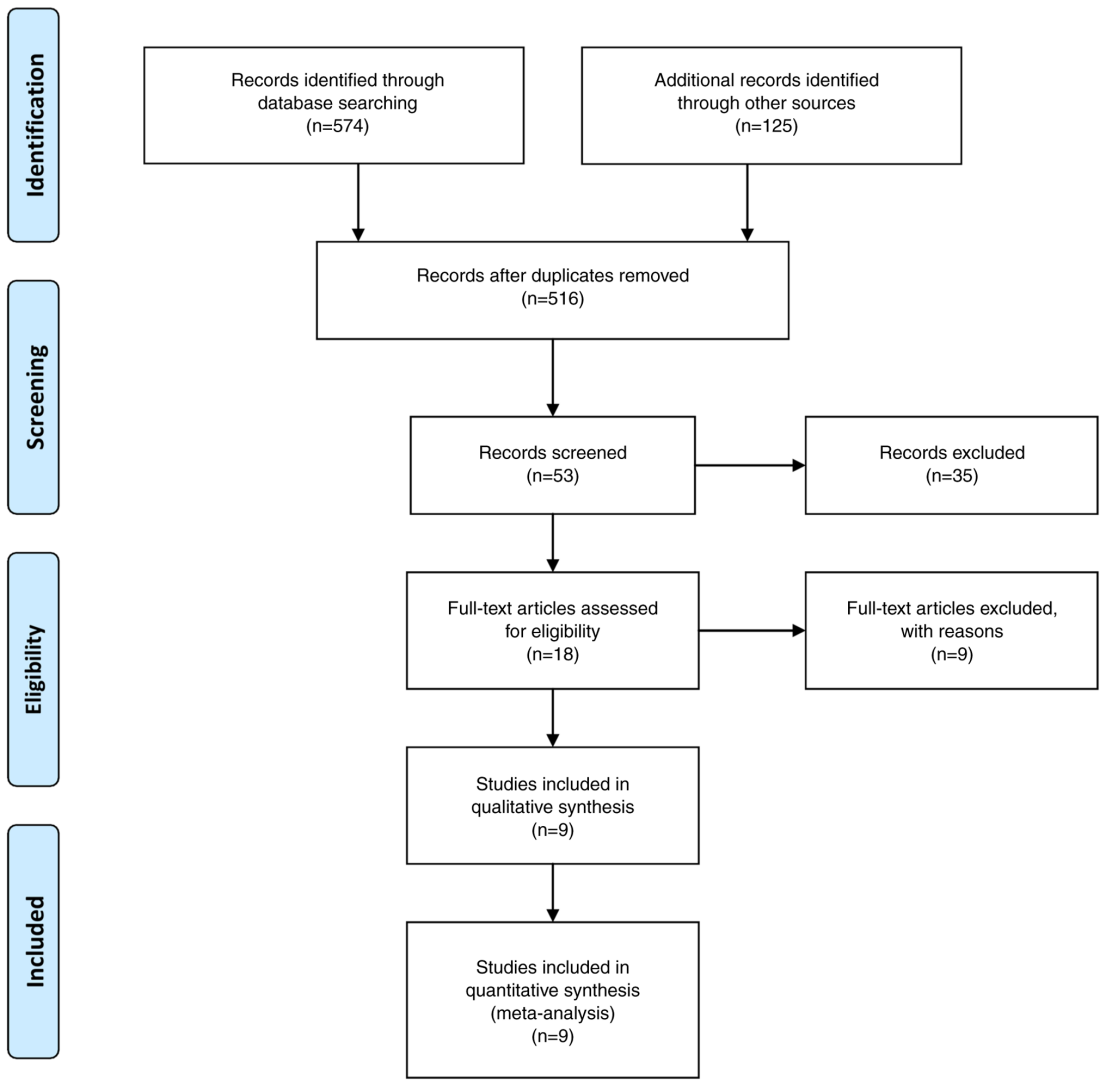
Flow chart of the study selection process in the present meta-analysis.

Following the primary search, 18 studies were suitable for further evaluation. When all the criteria were applied, nine articles were contained in the final study pool (Fig. 1) (3,11-18). The comprehensive data on these articles are presented in Table II. The total sample of patients collected from these nine articles was 3,253 (2,662 in the anterior and 591 in the posterior circulation UIAs group).

### Good recovery

**Figure 2:**
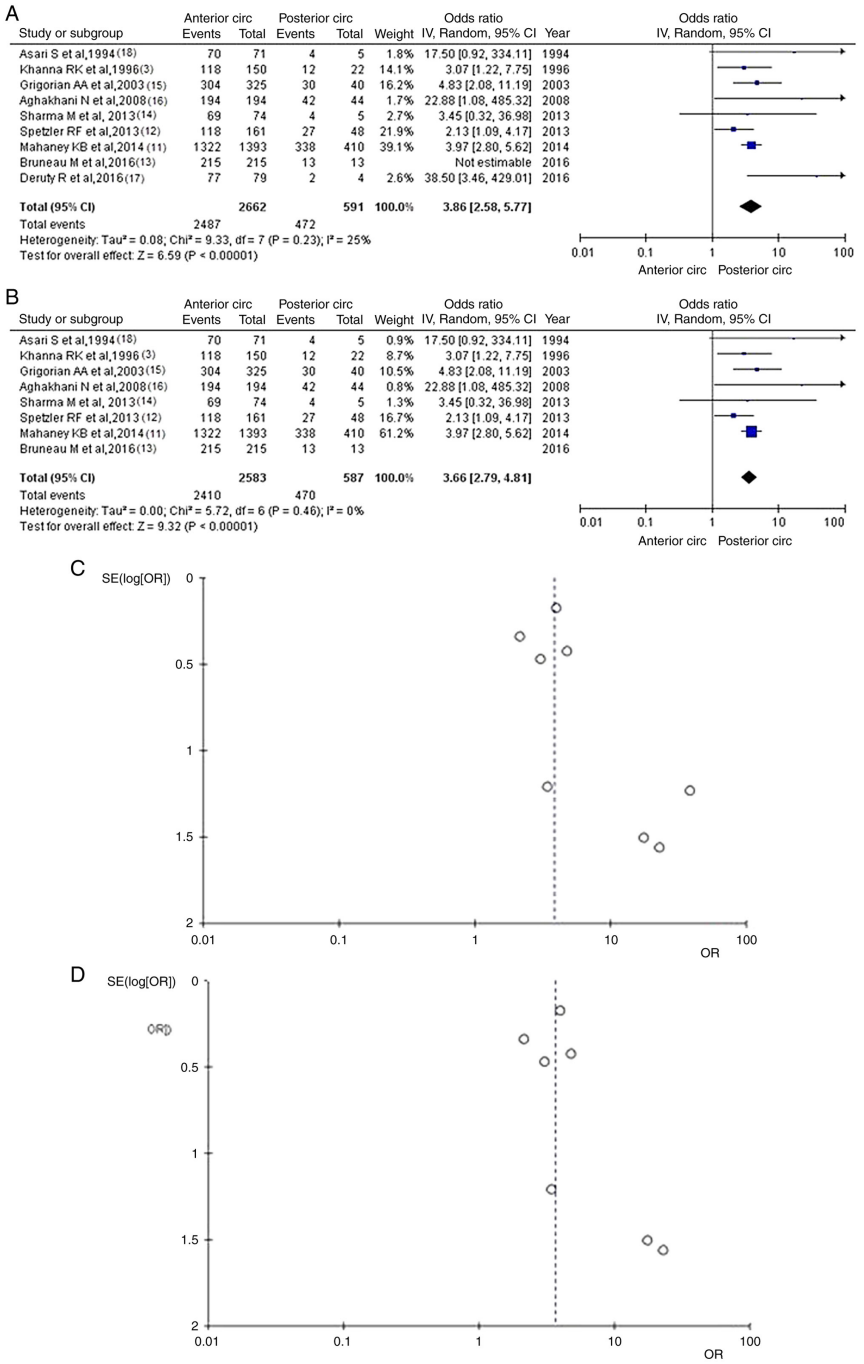
(A) Forest plot for good recovery: The results demonstrate a statistically significant difference between groups (OR 3.86; 95% CI, 2.58 to 5.77; P<0.05), with a low heterogeneity. (B) OR forest plot for good recovery without the study by Deruty *et al* (17). The results demonstrate a statistically significant difference (OR, 3.66; 95% CI, 2.79 to 4.81; P<0.05). (C) Funnel plot of good recovery between groups, with the study by Deruty *et al* (17) and with a low heterogeneity (P=0.23 and I^2^=25%). (D) Funnel plot of good recovery between groups, without the study Deruty *et al* (17), and without heterogeneity (P=0.46 and I^2^=0%). I^2^, the percentage of total variation across studies that is due to heterogeneity rather than chance; CI, confidence interval; OR, odds ratio.

A total of nine articles (3,11-18) provided information on good recovery following surgical treatment. There were 2,959 patients (2,487 or 93.42% in the anterior circulation group and 472 or 79.86% in the posterior circulation group), and there was a statistically significant difference between groups (OR, 3.38; 95% CI, 2.58 to 5.77; P<0.05), demonstrating the statistical superiority of the anterior circulation group of UIAs; however, there was low heterogeneity (P=0.23 and I^2^=25%) (Fig. 2A). While evaluating the sensitivity, one study was removed at a time using the ‘leave-one-out’ model (Table III). Following the removal of the article by Deruty *et al* (17), there was additionally a statistically significant superiority over the groups (OR, 3.66; 95% CI, 2.79 to 4.81; P<0.05), with no heterogeneity (P=0.46 and I^2^=0%) (Fig. 2Β). When the funnel plot was utilized for the analysis of the same parameter, it was found that the study results without the study by Deruty *et al* (17) revealed a better dispersion with no publication bias compared with the results of the same analysis if this one article was included (Fig. 2C and D).

### mRS >2

**Figure 3:**
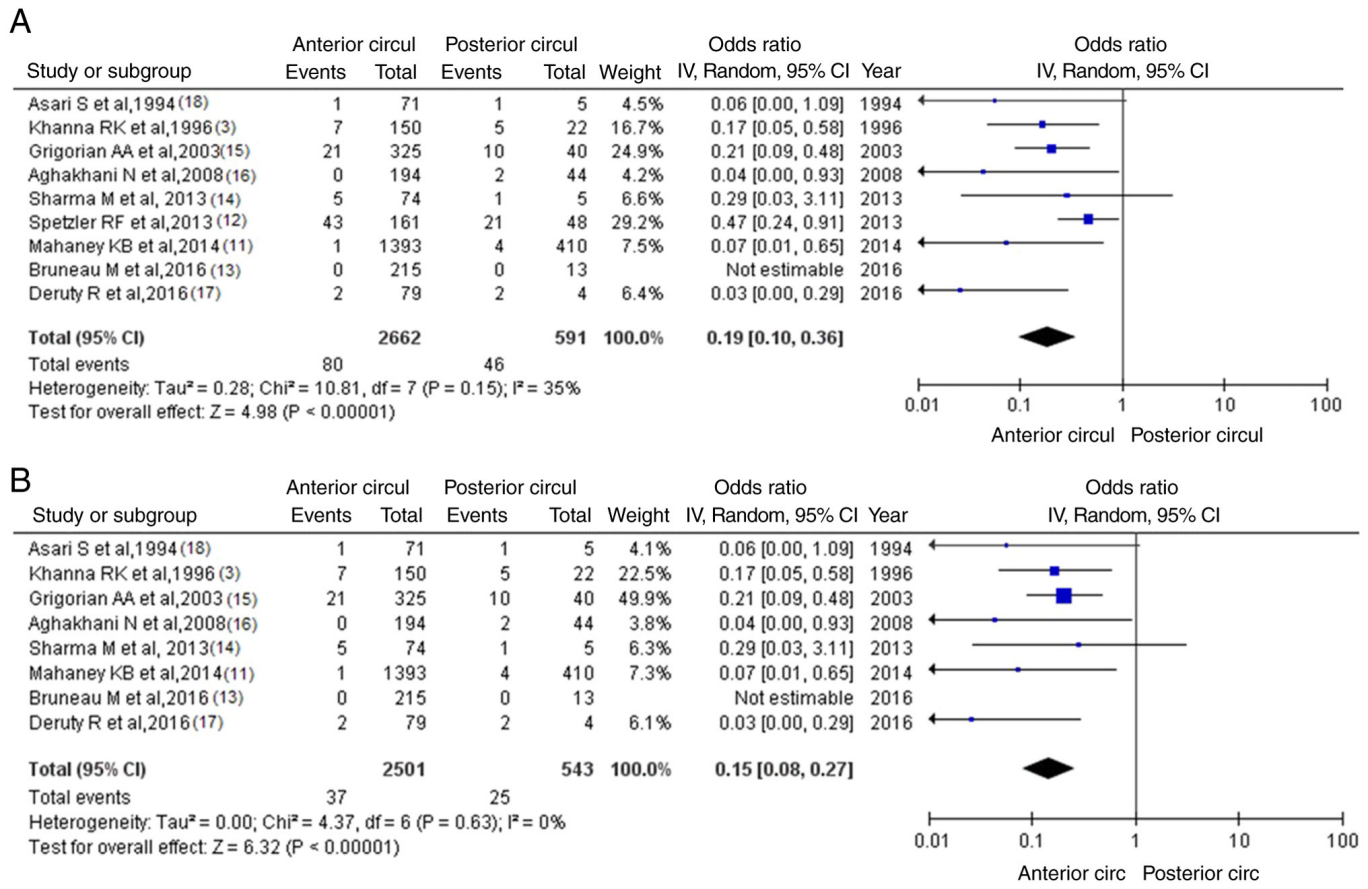
(A) Forest Plot of mRS >2. The results demonstrate a statistically significant difference between groups (OR, 0.19; 95% CI, 0.10 to 0.36; P<0.05), but with a low heterogeneity. (B) OR forest plot for mRS >2 without the study by Spetzler *et al* (12) article. The results again demonstrated no statistically significant difference (OR, 0.15; 95% CI, 0.08 to 0.27; P<0.05). I^2^, the percentage of total variation across studies that is due to heterogeneity rather than chance; CI, confidence interval; OR, odds ratio.

**Figure 4:**
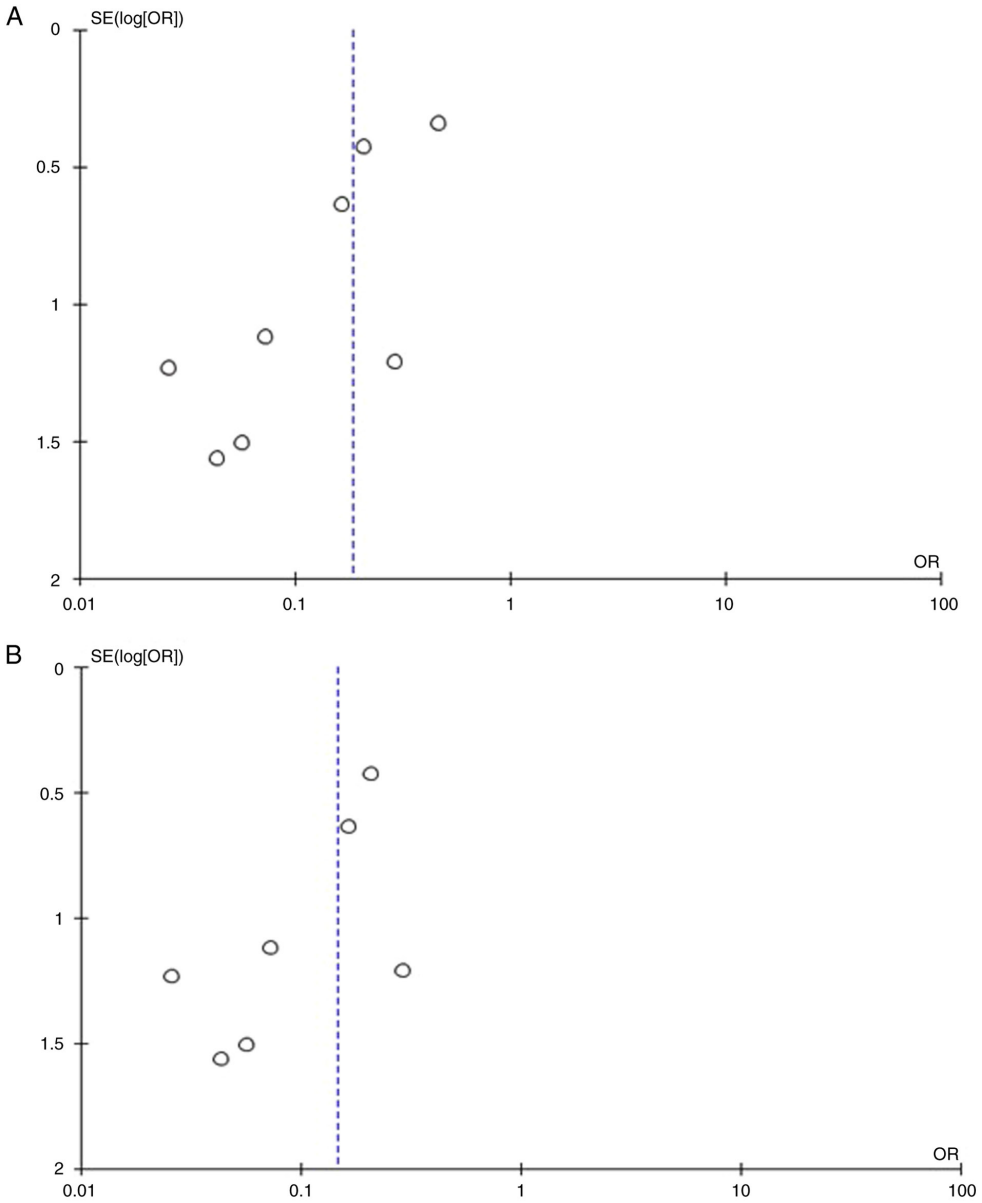
(A) Funnel plot of the mRS >2 parameter between groups, with the study by Spetzler *et al* (12), and with heterogeneity (P=0.15 and I^2^=35%). (B) Funnel plot of the mRS >2 parameter between groups, without the study by Spetzler *et al* (12), and a low heterogeneity (P=0.63 and I^2^=0%). mRS, modified Rankin scale; I^2^, the percentage of total variation across studies that is due to heterogeneity rather than chance; CI, confidence interval; OR, odds ratio.

Information regarding mRS >2 was available in nine articles (3,11-18). There were 126 patients (80 or 3.00% in the anterior circulation group and 46 or 7.78% in the posterior circulation group), and there was a statistically significant difference between groups (OR, 0.19; 95% CI, 0.10 to 0.36; P<0.05), demonstrating the statistical superiority of the anterior circulation group of UIAs; however, there was a low heterogeneity (P=0.15 and I^2^=35%) (Fig. 3A). While assessing the sensitivity, one study was removed at a time using the ‘leave-one-out’ model (Table III). After eliminating the article by Spetzler *et al* (12), there was additionally a statistically significant superiority over the groups (OR, 0.15; 95% CI, 0.08 to 0.27; P<0.05), with no heterogeneity (P=0.63 and I^2^=0%) (Fig. 3Β). When studying the funnel plot of the same parameter, it was observed that the study results without the study by Spetzler *et al* (12) revealed better dispersion with no publication bias, in contrast to the same analysis including this one article (Fig. 4A and B). Given that the patients in the study by Spetzler *et al* (12) represented 50.7% (64/126) of the included articles, this was not a surprise.

### Mortality

**Figure 5:**
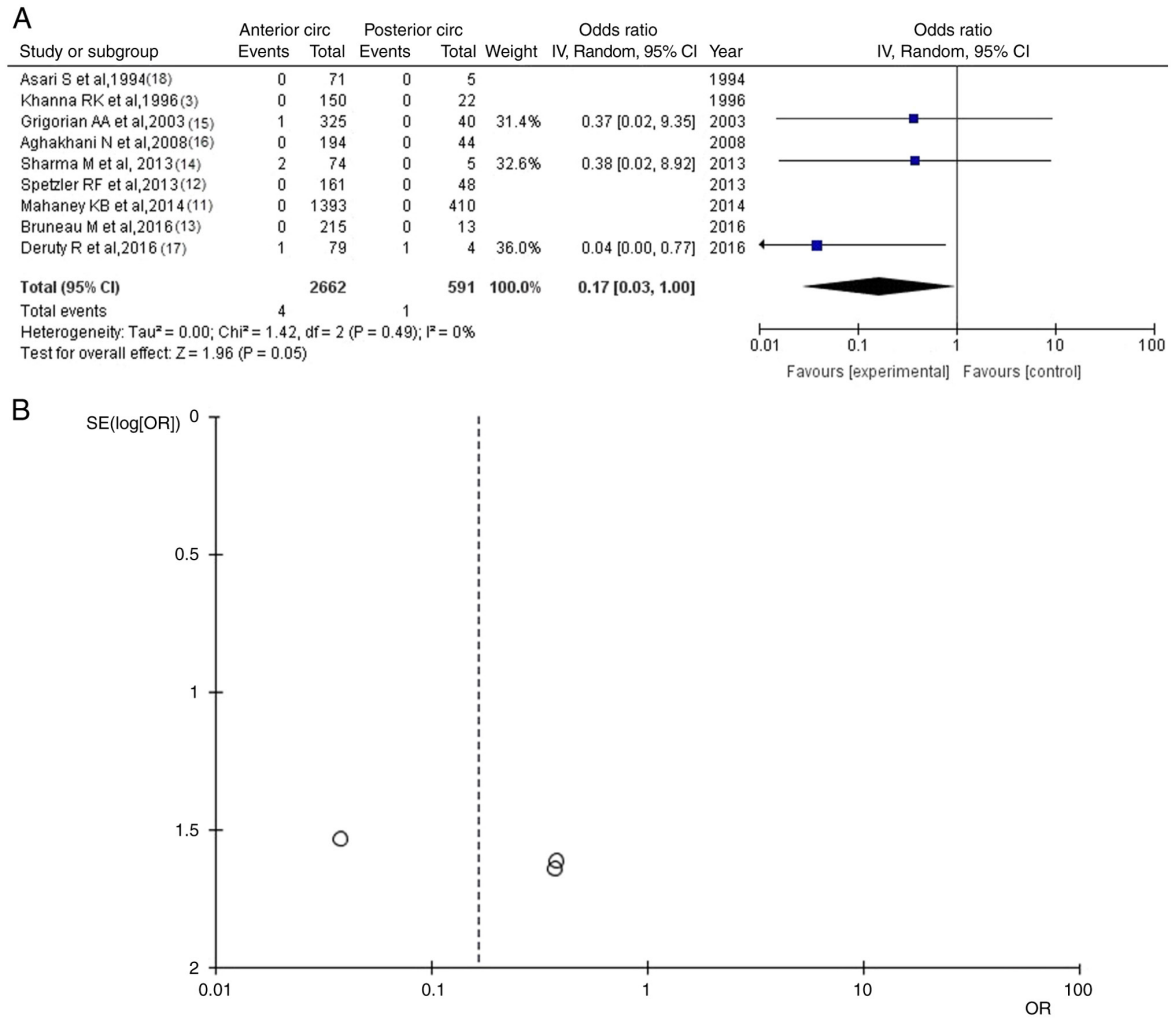
(A) OR forest plot for mortality. The results demonstrated a statistically significant results (OR, 0.17; 95% CI, 0.03 to 1.00; P=0.05). (B) Funnel plot of mortality in groups; the results demonstrated no heterogeneity (P=0.49 and I^2^=0%). I^2^, the percentage of total variation across studies that is due to heterogeneity rather than chance; CI, confidence interval; OR, odds ratio.

Information for mortality was available in nine articles (3,11-18). In the entry group of patients, there were 5 patients [4 (0.15%) in the anterior circulation group and 1 (0.17%) in the posterior circulation group], demonstrated a statistically significant difference between the groups (OR, 0.17; 95% CI, 0.03 to 1.00; P=0.05), with no heterogeneity (P=0.49 and I^2^=0% (Fig. 5A) and the superiority of the anterior circulation group compared with the posterior circulation UIAs group. A summary of the results of the present meta-analysis is presented in Table III.

**Figure 6:**
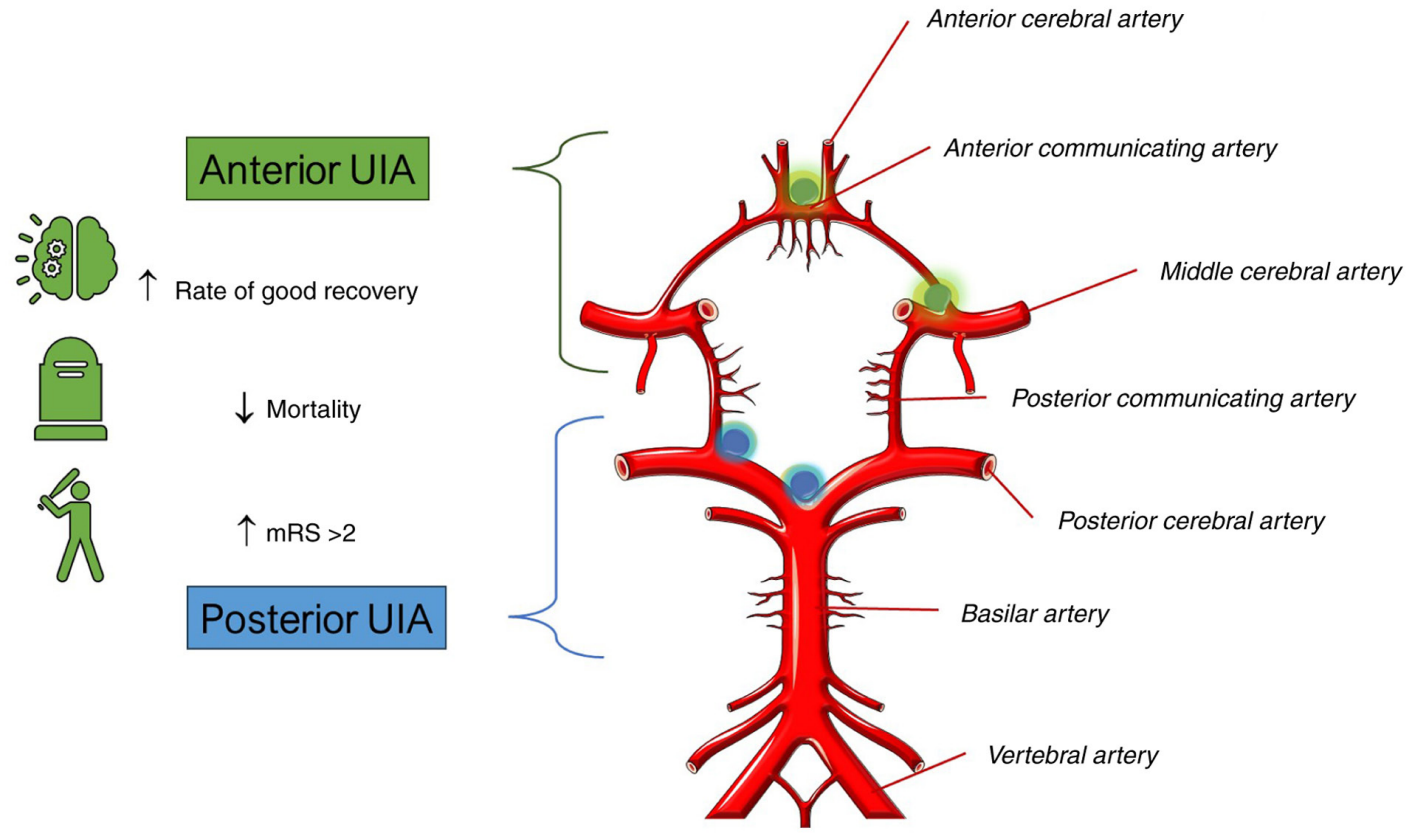
Summary of the results of the meta-analysis comparing the outcomes of surgical treatment for UIAs in the anterior and posterior circulation. Surgical treatment of anterior circulation UIAs is associated with better outcomes compared to posterior circulation UIAs. Major arteries and common sites of formation of intracranial aneurysms are also shown. mRS, modified Rankin scale. Parts of this image are derived from the free medical site http://smart.servier.com/ (accessed on 18 October 2023) by Servier, licenced under a Creative Commons Attribution 3.0 Unported Licence. UIAs, unruptured intracranial aneurysms.

A summary of the meta-analysis results comparing the outcomes of surgical treatment for UIAs in the anterior and posterior circulation is presented in Fig. 6.

## Discussion

Τhe optional modality for UIAs was to treat them (4,5); however, that recommendation is applied mostly for anterior circulation aneurysms, while the management method of a posterior circulation aneurysm is a debatable issue (3).

Thus, the present meta-analysis proposes that the surgical treatment of the anterior circulation UIAs is associated with better outcomes than the surgical management of posterior circulation UIAs. More precisely, mortality was a statistically significant parameter in patients with UIAs who were surgically treated, demonstrating the superiority of anterior compared to posterior circulation UIAs. In addition, mRS >2 and good recovery were statistically significant factors, demonstrating the advantage of surgical management of the anterior circulation UIAs compared with posterior circulation UIAs.

It has been reported that hemorrhage rates are significantly higher in the untreated group than in surgically treated patients (3). However, derived from a previously identified natural history between posterior vs. anterior circulation aneurysms, anterior circulation aneurysms hemorrhage less frequently (3). In addition, UIAs with posterior circulation aneurysms have 0.5% 1-year hemorrhage rates and morbidity. Of note, the hemorrhage rates and morbidity for patients >65 years of age with UIAs have been shown to not differ significantly by surgical management (3). On the other hand, in the same study and for the same subgroup of patients with an aneurysm size >13 mm, 33% of procedure-related morbidity was reported (3). In the present meta-analysis, the morbidity was twice higher in posterior compared with anterior circulation UIAs.

Other studies accounting for outcomes following surgery for UIAs have established 0 to 18% morbidity and 0 to 4% mortality (3); however, these studies did not include posterior circulation aneurysms, possibly due to the high risk of morbidity related to their surgical treatment (11,19-21). On the other hand, Drake *et al* (22) reported a 14.3% morbidity rate with the surgical management of UIAs in the posterior circulation compared to 0% morbidity in anterior circulation UIAs. However, the results of the present meta-analysis confirm the prognostic significance of aneurysm location for surgical outcomes. In effect, patients with an aneurysm in the posterior circulation had an almost 2-fold higher risk of an unfavorable outcome following surgical management than those with an aneurysm in the anterior circulation. Posterior circulation and aneurysms in difficult-to-access areas (arachnoid aneurysms, cavernous internal carotid artery) are possibly technically complex for representation and clip. They may have an increased morbidity and mortality associated with their treatment. Thus, the aneurismal location affects the operative morbidity. Even though limited data are available on the surgical treatment of UIAs of the posterior circulation exists, in the accommodating study (23), patients with UIAs in the anterior circulation had surgical morbidity rates between 4.8 and 16.8%. In addition, research has mentioned the high surgical risk of UIAs sited on the vertebrobasilar artery (24). However, unruptured aneurysms of the posterior circulation can be surgically treated with a low operative risk (25). The International Study of Unruptured Intracranial Aneurysms (ISUIA) recorded the overall morbidity and mortality in microsurgically treated patients at 1 year as 12.6%, counting cognitive impairment (8) and the evaluated risk factors as possible interpreters of the outcome. However, the ISUA included a larger number of patients with large aneurysms, a larger sum of patients with posterior communicating artery and posterior circulation aneurysms, and the ISUIA had 12.4% cavernous aneurysms, which are known to have a more benign course (8). In addition, a previous meta-analysis on the outcomes of surgery for unruptured aneurysms, including studies from 1966 to 1996, mentioned a mortality rate of 2.6% and a morbidity of 10.9%. Still, compared with the present meta-analysis, the majority of the involved studies did not include novel neurosurgical techniques or equipment and analyzed separated anterior and posterior circulation UIAs as surgical treatments. Thus, there is a risk of bias (26).

The majority of comparable studies and reviews refer to non-randomized studies (8,11) and have found no direct facts of clinical benefit from either treatment concerning the natural history of these lesions, raising a dilemma for both patients and neurosurgeons. Furthermore, patients with unruptured intracerebral aneurysms <7 mm in size with no evidence of rupture have been shown to have a very low bleeding rate (0 to 1% per year) (8,11). Consequently, obtaining a better natural history of these aneurysms would be challenging.

A number of considerations are used in the management of patients with UIAs. Patients <50 years of age with aneurysms that are ≤20 mm or less in the anterior circulation have better surgical outcomes. By contrast, patients >50 years of age, particularly those with large aneurysms in the posterior circulation, have the lowest surgical morbidity (27). Other key topics that require assessment include the patient's age (e.g., to establish whether the older patient has a worse outcome), aneurysm size, location (posterior and anterior circulation), history of stroke (major stroke is related to the poorest outcome), sex (female vs. male) and the duration of hospital stay.

In many studies for overall management, it has been established that posterior circulation aneurysms have the poorest outcome compared with anterior circulation, which was the case for both microsurgically and coiled-treated patients (8,14,26). On the other hand, further analysis in a number of types of research has not succeeded in demonstrating a statistically significant difference in the outcome of surgically managed aneurysms when evaluating anterior and posteriorly located aneurysms, even though this relation was preserved for coiled-treated aneurysms (26). The current year's modifications to aneurysm management training standards may help to explain this. Posterior aneurysms were treated more commonly with endovascular procedures compared with microsurgical intervention; as a consequence of the diversion of possible unfavorable outcomes, posterior aneurysms avoided surgical intervention, and on the way to endovascular management, morbidity for the comparatively small number of posterior aneurysms in the microsurgical group of patients revealed a minimal difference in outcomes compared with the anterior lesions. Additional patients need to be studied before any statistical significance can be reached. However, in the present meta-analysis, a tendency towards improved outcomes for patients with anterior circulation aneurysms undergoing microsurgery was observed.

Studies indicate that large aneurysms in the posterior region are more likely to hemorrhage, while small ones in the anterior circulation are less likely to hemorrhage. Even though this information should be considered when treating patients with UIAs, the majority of neurosurgeons cannot disregard the fact that several studies with ruptured aneurysms indicate that small-sized lesions were the most frequent aneurysms to rupture (28-30). This generates a question for physicians who are ambiguous about what they face in their everyday practice and what is being published in the literature. This is more complex, as the majority of patients with a history of aneurysm rupture may not be admitted to the hospital, and another 25% experience severe permanent brain injury. In addition, it appears to be a very challenging case for the treating neurosurgeon to decide for a young patient with a small and unruptured aneurysm. In this challenging decision-making situation, the neurosurgeon has to take into account the fact that it is a very superficial thought that the location and size of an aneurysm are sufficient data with which to make a serious choice in forecasting the performance of an aneurysm (26).

On the other hand, it must be considered that patients who undergo surgery for UIAs from the anterior or posterior circulation may experience retained strokes or hemorrhages on the additional follow-up. However, if we pay attention to a complete aneurysm clipping, it is enormously doubtful that it will be the reason for such strokes or novel hemorrhages. However, if all the possible locations (anterior or posterior) and other reasons for poor outcomes that could influence a certain population are taken into account, this would lead to an enormous amount of probability, from the inherent characteristics of each patient to their type of nutrition habits. It should be recognized that, even though statistics need calibration, medicine necessitates much perception, and the reality is that statistical results include several probabilities in the best case, while medical management requires diligent conclusions.

There are several limitations to the present study. First, the majority of the eligible reports that were included were retrospective. These retrospective studies, by definition, rely on imprecision and data loss. Additionally, the methods of the included studies markedly differed. Among these differences was the length of follow-up (e.g., 30-90 days). A longer follow-up period with these patients is warranted in order to correctly set up outcomes associated with treatment procedures. Additionally, the present study did not address outcomes in patients with unruptured aneurysms that are managed conservatively.

In conclusion, the present study demonstrates that the surgical treatment of patients with anterior circulation UIAs is associated with better outcomes than the surgical management of posterior circulation UIAs. In fact, mortality was a statistically significant parameter in patients with UIAs who were surgically treated, exhibiting the superiority of anterior compared to posterior circulation UIAs. In addition, mRS >2 and a good recovery were statistically significant factors, demonstrating the advantage of surgical management of the anterior circulation UIAs more than the posterior circulation. These findings indicate that surgical treatment may benefit the management of anterior circulation UIAs. It is also beyond doubt that a randomized trial is required in order to determine the difference in outcomes between these two treatment modalities in these patients.

## Figures and Tables

**Table I:** Newcastle-Ottawa scale quality assessment of the final article pool.

		Newcastle-Ottawa scale				
Authors, year of publication	Study design	Selection	Comparability	Exposure	Total scores	(Refs.)
Asari and Ohmoto, 1994	Retrospective	3	3	3	9	(18)
Khanna *et al*, 1996	Retrospective	3	2	2	7	(3)
Grigorian *et al*, 2003	Prospective	3	3	3	9	(15)
Aghakhani *et al*, 2008	Retrospective	3	2	2	7	(16)
Sharma *et al*, 2013	Retrospective	3	2	2	7	(14)
Spetzler *et al*, 2013	Prospective	3	3	3	9	(12)
Mahaney *et al*, 2014	Retrospective and prospective	3	3	3	9	(11)
Bruneau *et al*, 2016	Prospective	3	2	2	7	(13)
Deruty *et al*, 2016	Retrospective	3	2	2	7	(17)

**Table II:** Design and baseline characteristics of the included studies.

	Sample size				Anterior circulation			Posterior circulation			Size		Good recovery		mRS >2		Mortality		
Authors, year of publication	Ant. circ	Post. circ	Mean age (years)	No. of males	Acom	MCA	ICA	Pcom/PCA	PICA/SCA	Basilar type	<19 mm	>19 mm	Ant. circ	Post. circ	Anterior Circ	Post. circ	Ant. circ	Post. Circ	(Refs.)
Asari and Ohmoto, 1994	71	5	61.5	36	13	29	29	1	-	4	68	8	70	4	1	1	0	0	(18)
Khanna *et al*, 1996	150	22	51.9	50	-	-	-	-	-	-	140	32	118	12	7	5	0	0	(3)
Grigorian *et al*, 2003	325	40	53.5	NR	51	101	173	-	-	40	159	206	304	30	21	10	1	0	(15)
Aghakhani *et al*, 2008	194	44	49	127	44	117	33	40	3	1	178	60	194	42	0	2	0	0	(16)
Sharma *et al*, 2013	74	5	55.07	21	14	28	3	9	4	0	69	10	69	4	5	1	2	0	(14)
Spetzler *et al*, 2013	161	48	NR	NR	75	29	20	39	23	8	209	0	118	27	43	21	0	0	(12)
Mahaney *et al*, 2014	1,393	410	52.7	NR	285	615	493	304	-	106	701	407	1,322	338	1	4	0	0	(11)
Bruneau *et al*, 2016	215	13	51.3	55	22	137	4	1	7	5	NR	NR	215	13	0	0	0	0	(13)
Deruty *et al*, 2016	79	4	46	40	13	29	37	-	-	4	NR	NR	77	2	2	2	1	1	(17)

Ant. circ, anterior circulation; Post. circ, posterior circulation; Acom, anterior communicating artery; MCA, middle cerebral artery; ICA, internal carotid artery; PICA, posterior inferior cerebellar artery; SCA, superior cerebellar artery; mRS, mRS, modified Rankin scale.

**Table III:** Outcomes of the meta-analysis.

			Groups		Overall effect			Heterogeneity		
Parameters	‘Leave-one-out’ model	Trial, n=9	Ant. circ	Post. circ	Effect estimate	95% CI	P-value	I^2^ (%)	P-value	(Refs.)
Good recovery	-	9	2662	591	3.86	(2.58-5.77)	<0.05	25	0.23	
	Asari and Ohmoto, 1994	8	2591	586	3.75	(2.50-5.63)	<0.05	28	0.22	(18)
	Khanna *et al*, 1996	8	2512	569	4.14	(2.53-6.79)	<0.05	34	0.17	(3)
	Grigorian *et al*, 2003	8	2337	551	3.79	(2.30-6.26)	<0.05	33	0.18	(15)
	Aghakhani *et al*, 2008	8	2468	547	3.73	(2.52-5.53)	<0.05	25	0.24	(16)
	Sharma *et al*, 2013	8	2588	586	3.94	(2.52-6.17)	<0.05	36	0.16	(14)
	Spetzler *et al*, 2013	8	2501	543	4.22	(3.14-5.68)	<0.05	0	0.42	(12)
	Mahaney *et al*, 2014	8	1269	181	4.18	(2.24-7.83)	<0.05	34	0.17	(11)
	Bruneau *et al*, 2016	8	2447	578	3.86	(2.58-5.77)	<0.05	25	0.23	(13)
	Deruty *et al*, 2016	8	2583	587	3.66	(2.79-4.81)	<0.05	0	0.46	(17)
mRS >2	-	9	2662	591	0.19	(0.10-0.36)	<0.05	35	0.15	
	Asari *et al*, 1994	8	2591	586	0.19	(0.10-0.39)	<0.05	39	0.13	(18)
	Khanna *et al*, 1996	8	2512	569	0.18	(0.08-0.39)	<0.05	42	0.11	(3)
	Grigorian *et al*, 2003	8	2337	551	0.15	(0.06-0.38)	<0.05	43	0.10	(15)
	Aghakhani *et al*, 2008	8	2468	547	0.20	(0.10-0.39)	<0.05	37	0.14	(16)
	Sharma *et al*, 2013	8	2588	586	0.17	(0.08-0.36)	<0.05	44	0.19	(14)
	Spetzler *et al*, 2013	8	2501	543	0.15	(0.08-0.27)	<0.05	0	0.63	(12)
	Mahaney *et al*, 2014	8	1269	181	0.20	(0.10-0.40)	<0.05	37	0.14	(11)
	Bruneau *et al*, 2016	8	2447	578	0.19	(0.10-0.36)	<0.05	35	0.15	(13)
	Deruty *et al*, 2016	8	2583	587	0.24	(0.13-0.41)	<0.05	18	0.29	(17)
Mortality	-	9	2662	591	0.17	(0.03-0.77)	0.05	0	0.49	

Ant. circ, anterior circulation; Post. circ, posterior circulation; mRS, modified Rankin scale; I2, the percentage of total variation across studies that is due to heterogeneity rather than chance; CI, confidence interval.

## Data Availability

The datasets used and/or analyzed during the current study are available from the corresponding author on reasonable request.
